# Impact of online convenience on generation Z online impulsive buying behavior: The moderating role of social media celebrity

**DOI:** 10.3389/fpsyg.2022.951249

**Published:** 2022-08-03

**Authors:** You Lina, Deshuai Hou, Saqib Ali

**Affiliations:** ^1^Graduate Business School, UCSI University, Kuala Lumpur, Malaysia; ^2^School of Accounting, Capital University of Economics and Business, Beijing, China; ^3^Department of Management Sciences, COMSATS University Islamabad, Sahiwal, Pakistan

**Keywords:** online convenience, impulsive buying, social media celebrity, Gen Z, SOR model

## Abstract

This research aims to determine which dimensions of online convenience influence generation z consumers’ cognitive and affective attitudes and online impulsive buying behavior. The moderating effect of social media celebrity is also investigated to examine the attitude-behavior gap. A total of 348 responses from Chinese users who followed digital celebrities were received using purposive sampling. Data analysis and hypothesis testing were carried out using SmartPLS, version 3 (partial least squares). The results indicated that relationship convenience, possession convenience, post possession conveniences, transaction convenience, and evaluation convenience have a crucial impact on cognitive and affective attitudes. Similarly, cognitive and affective attitudes are significant and positive predictors of generation z consumers’ online impulsive buying behavior. Moreover, empirical results supported the moderating role of social media celebrities that aid marketers in selecting a product endorser. The implications for marketers and policymakers are discussed based on the above research findings. Moreover, future research directions are suggested.

## Introduction

An unexpected or non-essential purchasing action is known as impulse buying (Amos et al., 2014; Iyer et al., 2020; Bandyopadhyay et al., 2021; Cavazos-Arroyo and Máynez-Guaderrama, 2022; Nigam et al., 2022). It is rapid and spontaneous, with little consideration of the product or the repercussions of the purchase (Lin and Chen, 2013; Rezaei et al., 2016; Iyer et al., 2020). The phrase ‘impulse buying refers to a person’s proclivity to make unexpected, quick, and unreflective purchases (Sohn and Lee, 2017; Bandyopadhyay et al., 2021). Many scholars and practitioners have recently focused their attention on impulsive shopping behavior, which is sometimes referred to as “impulse shopping” (Aragoncillo and Orus, 2018; Kumar et al., 2020). Two-thirds of supermarket sales are accounted for by this category (Amos et al., 2014; Katakam et al., 2021). According to a recent study, an impulsive purchase may be classified into two thematic contexts: online and offline stores (Amos et al., 2014; Kimiagari and Malafe, 2021). Several investigations have shown that over 50% of internet purchases are done on impulse (Zheng et al., 2019). Impulsive buying is widespread around the globe (Bandyopadhyay et al., 2021). For example, It is claimed that 80% of American teens have made impulsive online purchases and that consumers in the United Kingdom (UK) spend around £1 billion each month on impulsive buying (Gulfraz et al., 2022). In emerging markets such as Pakistan (Miao et al., 2019), India (Ekambareswarar et al., 2021) and, more importantly, China (Gulfraz et al., 2022), impulsive purchasing is also on the rise. China’s e-shopping population surpassed 1 billion in 2019, with a total expenditure of $636.09 billion compared to American shoppers, who spent 258.5 million (Ampadu et al., 2022).

It has been shown that impulsive purchasing is common among Generation Z (those born between the mid-1990s and the early-2010s) due to the rapid growth of social networking sites (SNS) like Facebook, Twitter, and Pinterest (Priporas et al., 2017; Djafarova and Bowes, 2021; Liu et al., 2021; Zafar et al., 2021). In addition, as social media usage has grown, a new breed of celebrity has emerged, referred to as social media celebrity, i.e., social media influencers, Instafamous, vloggers, and bloggers. Social media celebrities are more credible and influential than traditional celebrities, i.e., movie stars, artists, athletes, and TV stars (Djafarova and Rushworth, 2017). Many individuals follow such digital celebs due to their authenticity (Djafarova and Rushworth, 2017; Djafarova and Bowes, 2021). These digital celebrities may impact a user’s impulsive buying behavior (Xiang et al., 2016). According to research, 41% of Generation Z customers are impulsive shoppers, followed by 34% of Millennials and 32% of Generation X (Djafarova and Bowes, 2021). Gen Z consumers are more persuasive while making purchases (Lee et al., 2022). They want new products faster (Agrawal, 2022). They are brand-less and fashion-hungry (Djafarova and Rushworth, 2017). Brands must build marketing tactics to reach this demographic (Djafarova and Bowes, 2021; Agrawal, 2022). Generation Z grew up on the Internet. Therefore they use social media for inspiration (Djafarova and Bowes, 2021).

One of the primary motivators driving impulsive consumer buying is online convenience (Gulfraz et al., 2022). As consumers devote less time to shopping and more to other activities, they want ease and turn to virtual shopping (Jiang et al., 2013; Duarte et al., 2018). Thus, it is necessary to understand the link between online convenience and impulsive buying to understand the consumer base and the e-commerce business. Academic research on online convenience as a stimulus for impulsive online shopping is rare, despite its expanding importance in consumer behavior (Jiang et al., 2013; Duarte et al., 2018; Shankar and Rishi, 2020; Jebarajakirthy and Shankar, 2021; Shankar, 2021; Shankar et al., 2021). An overview of the relevant studies may be found in Table 1.

**Table 1:** Summary of notable studies in the literature that capture online convenience.

Author	Constructs	Study context	Findings
Jiang et al. (2013)	Access, Search, Evaluation, Transaction, Possession, and post possession Convenience	Online Shopping	The findings provide more evidence that the convenience of online shopping can be broken down into five distinct categories: access, search, evaluation, transaction, and possession/post-possession.
Duarte et al. (2018)	Access, Search, Evaluation, Transaction, Attentiveness, Possession, and post possession convenience	Online Shopping	According to the study findings, the dimensions of convenience associated with online shopping that are most strongly influenced are “Possession,” “Transaction,” and “Evaluation.”
Shankar and Rishi (2020)	Access, Search, Evaluation, Transaction, Possession, and post possession Convenience	Mobile banking	The convenience regarding access, transactions, possession, and post-possession was the most critical factor in determining whether consumers intend to use mobile banking.
Shankar (2021)	Access, Search, Evaluation, Transaction, Benefit, and post benefit convenience	Webrooming	According to the findings, a consumer’s intention to webroom is significantly influenced by the ease of access, search, benefit, and post-benefit convenience.
Jebarajakirthy and Shankar (2021)	Access, Search, Evaluation, Transaction, Benefit, and post benefit convenience,	Mobile banking	According to the findings, the ease of access, transactions, benefits, and post-benefit convenience significantly influence consumers’ intentions to use mobile banking.
Shankar et al. (2021)	Decision, attentiveness, evaluation, Transaction, Possession, and post possession convenience	Showrooming	According to the findings, convenience dimensions such as attentiveness, evaluation, possession, and post-possession convenience play a key influence in the formation of customers’ desire to engage in showrooming behavior.

The current understanding of consumer online impulse buying behavior and the contributing factors, i.e., Online Convenience (Duarte et al., 2018) or social media celebrities (Djafarova and Bowes, 2021), is limited. We developed a model based on the Stimulus-Organism-Response (SOR) model (Mehrabian and Russell, 1974) to fill this knowledge gap. Theorizing different dimensions of online convenience, i.e., Access convenience, search convenience, transaction convenience, evaluation convenience, relationship convenience, benefit convenience and post benefit convenience as stimuli (S), employing bi-dimensional attitudes, i.e., cognitive and effective as an organism (O) and online impulsive buying behavior as a response (R). Frank-Martin and Peattie (2009) discovered that the attitude-behavior gap is a discrepancy between what individuals assert and convey *via* their attitudes and how they act. More study is needed to close the gap between attitude and behavior, which prevents individuals from turning their attitudes into behavior (Wiederhold and Martinez, 2018; Sadiq et al., 2022). This research attempts to answer that call by studying the moderating role of social media celebrity on the relationship between attitude and behavior.

In light of this, our research makes the following distinct contributions: (a) It is one of the limited studies to examine the dimension of convenience. (b) It is the first study to examine Gen Z impulsive buying in the theoretical backdrop of SOR with bi-dimensional attitude approach. (c) It is among the limited studies that have examined the moderation effect of social media celebrity on the association of attitude and behavior.

The present research is organized as follows: The review of relevant literature and the development of hypotheses are presented in “Literature review.” The research methodology is presented in “Materials and methods,” and the study findings are presented in “Data analysis and results. discussion,” theoretical and practical implications, limitations, and future research directions are all presented in “Discussion and implications.”

## Literature review

### Impulse buying

Unplanned or non-essential purchasing behavior is known as impulsive buying (Muruganantham and Bhakat, 2013; Aragoncillo and Orus, 2018; Sen and Nayak, 2021). It happens instantly and without rigorous assessment of the goods and purchase’s repercussions (Khachatryan et al., 2018; Lee et al., 2021; Bao and Yang, 2022). Impulsive buying is described by Beatty and Ferrell (1998) as any “sudden and instant purchase with no pre-shopping plans to purchase the particular product category or to complete a specified purchasing job.” The phrase ‘impulse purchasing propensity’ refers to an individual’s proclivity to make unexpected, quick, and unreflective purchases (Beatty and Ferrell, 1998; Khachatryan et al., 2018). Customers may quickly obtain information about goods or services *via* the Internet, which has become a fundamental part of everyday life. When compared to conventional shopping, internet shopping allows for greater impulsive purchases. The feasibility of social media, in particular, is a new way to boost marketing efforts considerably and may play a vital role in influencing customer purchasing choices, such as impulsive buying (Alalwan et al., 2017; Kapoor et al., 2018; Dwivedi et al., 2021). Understanding how people buy on impulse is essential for business. Sharma et al. (2010) argued that people shop online because of their emotions, spontaneous behavior, or lack of cognitive control. They also say that appealing objects cause people to act impulsively, making them buy things without thinking about financial or other aspects of online shopping. From this point of view, few scholars argue that people who buy products online are more impulsive than people who buy products in stores (Verhagen and Van Dolen, 2011; Park et al., 2012; Ozen and Engizek, 2014). Wu et al. (2015) stated that the online marketing stimuli make online buyers less risk-averse during their first search and make it easy for them to buy on the spot (Madhavaram and Laverie, 2004; Jeffrey and Hodge, 2007; Lo et al., 2016).

### Online convenience

More and more consumers are turning to internet shopping for its convenience since they have less time to devote to shopping and more time to pursue other interests (Shaqman et al., 2022). Consumers’ lack of free time encourages them to look for ways to save time and effort while making purchases (Gehrt et al., 1996). Copeland (1923) initially used the term “convenience” to describe the amount of time and effort required to purchase consumer goods. Consequently, the term “retail convenience” may be defined as customers’ time and effort expenses when shopping in a retail setting. These consumer resources of time and effort are identified in marketing literature as non-monetary costs that impact purchase behavior (Herrmann and Beik, 1968; Jacoby et al., 1976). As a result, retailers have been concentrating on delivering services that expedite and simplify the purchasing experience for customers (Chang and Polonsky, 2012). Retailers raise the value of their market offer by enhancing customers’ convenience to save time and effort through convenience improvements (Lloyd et al., 2014). Internet is presently a viable choice for customers who wish to save time and energy. People choose online retail formats because their lifestyles are often constricted due to rising professional responsibilities, limiting the amount of time available for everyday activities and prompting them to select retail formats that need the least time (Bhatnagar et al., 2000). Their priority is to complete the shopping process while using the least effort possible to receive the desired item (Lloyd et al., 2014).

According to existing empirical evidence, convenience is a critical factor in the interaction between consumers and service providers. Customers are more likely to stick around if there is an excess of convenience available (Lloyd et al., 2014; Lovelock and Patterson, 2015). In contrast, a lack of convenience has been a primary cause for them to leave (Keaveney, 1995; Seiders et al., 2007; Lovelock and Patterson, 2015).

Despite the significance of convenience, there is no widespread agreement on the factors that compose online convenience. Farquhar and Rowley (2009) argue that online convenience is merely a proxy for the resources customers are using rather than something inherent to their service. For others (Yale and Venkatesh, 1986; Berry et al., 2002; Seiders et al., 2007), convenience is a multidimensional notion. Even though convenience has multiple dimensions, no one agrees on what they are (Seiders et al., 2007; Reimers and Chao, 2014). Access, search, assessment, transaction, possession and post-possession convenience are the five dimensions of convenience defined by Jiang et al. (2013). Consumers are more likely to engage in impulsive online purchases if they can easily avail the abundance of service convenience in term of search, evaluation, transaction, relationship, possession, and post-possession convenience.

### Conceptual background

By employing, Mehrabian and Russell (1974) SOR framework, researchers may better understand how consumers make impulsive online purchases (Hashmi et al., 2019; Zheng et al., 2019). This study employed it as a theoretical framework to investigate the associations between the convenience elements and online impulsive buying behavior. The basic SOR framework has three components: (1) a stimulus: “a trigger that stimulates the consumer,” (2) an organism: “the internal assessment of the consumer,” and (3) a response: “the consequence of the consumer’s reaction to online impulse buying drivers and their interior assessments” (Chen and Yao, 2018; Zhang et al., 2018). In other words, the S–O–R proposes that an organism exposed to external stimuli would process and react to those stimuli uniquely. In the current study, elements of online convenience serve s stimuli influencing the consumers’ cognitive and affective attitude (organism) and deriving consumers’ online impulsive buying behavior (response). Stimuli are classified in the literature into two basic categories: object stimuli and social psychological stimuli. Complexity, consumption duration, and product qualities are all dealt with by object stimuli; social-psychological stimuli, on the other hand, are related to an individual’s surrounding environment. The online service convenience (OSERVCON) multidimensional model is used to determine the most relevant object and social psychological stimuli for impulsive online purchases. Three emotional states are outlined in the stimulus organism response (S-O-R) model (Mehrabian and Russell, 1974): pleasure, arousal, and dominance (PAD), which represents the organism. Due to the limited scope of PAD dimensions, numerous alternative constructs associated with internal states such as emotive, evaluative, cognitive and affective have been proposed in the literature (Fiore and Kim, 2007; Lee and Yun, 2015). In addition, Eroglu et al. (2001) postulated two internal states: cognition and affect. These two states have higher explanatory power than the preceding ones. Research shows that attitude scales are helpful for capturing and operationalizing one’s own internal states of cognition and emotion (Fiore and Kim, 2007). We use the bi-dimensional approach to operationalize attitude as either cognitive or affective.

Scholars argued that investigations that use attitude as an indicator of actual behavior should be taken care of (Wiederhold and Martinez, 2018). Misalignment between stated attitude and actual behavior implies that buyers’ stated attitude and actual behavior are not always in sync at the time of purchase (Auger and Devinney, 2007; Wiederhold and Martinez, 2018). This phenomenon is known as the “attitude-behaviour gap.” (Davies et al., 2002; Carrington et al., 2010, 2014).

Based on the above discussion, this study investigates the influence of service convenience dimensions, i.e., assess convenience, search convenience, benefit convenience, post-benefit convenience and relationship convenience on the two dimensions of attitude: cognitive and affective. Moreover, moderating the role of social media celebrities is also investigated to improve the attitude-behavior gap. This moderating effect is crucial because many consumers seek social media personalities’ opinions before buying a product.

### Access convenience

This dimension is defined as “the speed and convenience with which customers may approach a store” (Shankar and Rishi, 2020). Access convenience is a crucial aspect of retail convenience since if the customer cannot reach the shop, he/she cannot use the service. In conventional retail, access convenience might be improved by shifting the store location (Seiders et al., 2007). In the internet context, store location is immaterial (Jebarajakirthy and Shankar, 2021). King and Liou (2004) indicated that website accessibility is the most significant aspect of online shopping convenience. This may be achieved by employing more user-friendly and easy-to-remember URLs, automated bookmarking technologies, and strategically positioning adverts on social networking sites. On this basis, it is argued that:

*H1*: Access convenience is positively associated with attitude towards online impulse buying behavior.

### Search convenience

Katawetawaraks and Wang (2011) state search convenience as “how quickly and easily customers find and choose things to purchase.” The Internet has provided numerous tools that have enabled retailers to enhance their communication with prospective customers by bolstering their ability to provide detailed information, either by integrating it into their website and using paid advertising to redirect traffic or by spreading information and generating buzz on social media, and hence assisting them in identifying and selecting the most suitable business partners (Beauchamp and Ponder, 2010). These upgraded technologies help customers avoid wasting time by avoiding crowds, minimizing waiting time, and going to physical shops (Katawetawaraks and Wang, 2011). Let us assume that a store’s more successful is in enabling consumer product searches. The customer’s trip through the purchasing experience will be faster and easier; the following hypothesis is proposed:

*H2*: Search convenience is positively associated with attitude towards online impulse buying behavior.

### Evaluation convenience

The availability of extensive but easy-to-understand product descriptions utilizing different presentation techniques, such as text, images, and video, on the company’s website is linked to evaluation convenience (Tankovic and Benazic, 2018). Consumers may acquire a sound vision of items, zoom and rotate them, alter colors, and declare how the products could match their requirements using these tools (Tran and Strutton, 2020). They may also compare costs and participate in online conversations with other shoppers (Shankar and Rishi, 2020). This form of product exposure helps consumers to compare products and make quick purchases. In recent years, the abundance of products and rich information has made online customers more sensitive than ever to the efforts connected with evaluation convenience (Jiang et al., 2013; Shankar and Rishi, 2020). So, here’s the hypothesis:

*H3*: Evaluation convenience is positively associated with attitude towards online impulse buying behavior.

### Transaction convenience

Transaction convenience is “how quickly and easily customers can make or change transactions” (Lovelock and Patterson, 2015). Online customers conduct transactions in “virtual checkout lines.” stores with 1-Click checkouts and simple returns are transactionally convenient. Online shoppers never have to wait in line (Shankar and Rishi, 2020). Privacy issues and unsafe transactions may discourage internet purchases. Customers need secure, easy online payment solutions (Jebarajakirthy and Shankar, 2021). According to Shankar and Rishi (2020), the fear of losing money and financial information deters people from purchasing online. Hence, it is argued that:

*H4*: Transaction convenience is positively associated with attitude towards online impulse buying behavior.

### Relationship convenience

Due to increased competition in online markets, merely exposing product or service catalogues on the Web is not enough to assure online business longevity. The relationship convenience relates to how many online retailers deliver individualized services and attention to their clients (Zahid et al., 2022). Online shoppers demand individualized services to decrease the time and effort required to seek information and make a purchasing choice (Lovelock and Patterson, 2015). Modern online retailers use personalization features to distinguish their goods and services from the competition (Zahid et al., 2022), boosting consumer experience and convenience. Customers may comprehend information more quickly and fluently using online personalization capabilities, which leads to increased purchasing experience (Lovelock and Patterson, 2015). Online merchants provide decision aids (i.e., suggestion agents or shopping bots) and even human assistants to ease purchasing choices and improve consumer experience (Christopher et al., 2013). Researchers hypothesize that:

*H5*: Relationship convenience is positively associated with attitude towards online impulse buying behavior.

### Possession convenience

Possession convenience is the time and money customers expend to get what they want (Jiang et al., 2013). Possession convenience is the quickness and simplicity with which customers may receive desired items, including production planning, stoking policies, shipping, and delivery schedules. Traditional retailers have the benefit of allowing you to depart with the thing you want (McKinney et al., 2002; Ganesh et al., 2010). In online retailers, purchasers must wait for their goods to be processed, shipped, and delivered. Time spent completing all stages of the online purchase procedure and waiting for delivery might be considered a non-monetary cost of doing business online (Jiang et al., 2013). According to Islam (2015), worries regarding order delivery affect online shopping. So, here’s the hypothesis:

*H6*: Possession convenience is positively associated with attitude towards online impulse buying behavior.

### Post-possession convenience

Post-possession convenience is “the consumer’s perceived time and effort expenditures when re-contacting a firm after acquiring the intended goods” (Lovelock and Patterson, 2015). In recent years, post-possession convenience has been stressed due to problems returning online purchases (Lovelock and Patterson, 2015). Post-possession convenience factors frequently relate to product repair, maintenance, or exchange (Rust and Oliver, 1993). Other factors, such as transaction issues, customer complaints, guarantee fulfilment, or faulty items or services, may cause consumers to adjust their online convenience rating (Jiang et al., 2013). Online convenience increases as users spend less time and effort dealing with broken services. So, here’s the hypothesis:

*H7*: Post-possession convenience is positively associated with attitude towards online impulse buying behavior.

### Attitude and online impulse buying behavior

Attitude is described as a broad, enduring, and ongoing assessment of a person, place, or thing. The term “attitude” refers to a person’s positive or negative feelings towards an object. Attitude toward a behavior is the degree to which someone likes or dislikes the behavior in the question. Attitude is a multidimensional construct that includes cognition, affect, emotion, value, and awareness. Following Eroglu et al. (2001) taxonomy of attitude, we employed two dimensions of attitude in our study: cognitive attitude and effective attitude. Cognitive attitude is how much a person likes or dislikes an object based on how useful it is and what functions it performs (Fiore and Kim, 2007; Celebi, 2015). A person’s affective attitude is made up of the feelings and sensations that come from using or experiencing an object (Fiore and Kim, 2007).

Novak et al. (2003) think both cognitive and emotional attitudes impact online impulsive purchases. Babin et al. (2004) and Zheng et al. (2019) verified that cognitive and emotional dimensions are complimentary and positively related. The previous study has also shown a link between cognitive and affective attitudes and impulsive online purchases (Kim and Eastin, 2011; Verhagen and Van Dolen, 2011; Zheng et al., 2019).

*H8*: Attitude towards online impulse buying behavior is positively associated with online impulse buying behavior.

### The moderating role of social media celebrity

Thanks to social media’s infinite digital environment, individuals may freely create content and interact with enormous audiences. As user-generated content increased on social media, individuals’ duties shifted from passive receivers to proactive generators/distributors of market information (Lee and Eastin, 2021). Furthermore, the interactive features of social media enable specific individuals to build massive networks through which they may influence other users. These individuals are known as social media celebrities (SMCs) and act as opinion leaders for a broad audience (Casaló et al., 2020). There are two types of celebrities: traditional and non-traditional. Traditional celebrities include actors, singers, sports, and T.V. personalities. Non-traditional celebrities include bloggers, YouTube stars, and social media personalities. Users want to follow such celebrities because they are authentic, and it has gotten more attention in the marketing world (Moulard et al., 2015; Audrezet et al., 2020). Online celebrities are considered more trustworthy than traditional celebrities (Djafarova and Rushworth, 2017; Djafarova and Bowes, 2021). According to Nouri (2018), the information provided by an online star is considered more genuine and impactful. Several fields have featured celebrities, including lifestyle, entertainment, and cuisine (Kumar and Mirchandani, 2012; Zoha, 2018; Bradri, 2019). These celebrities have accounts, groups, or websites on different social media platforms where they share their shopping experiences (Kawasaki and Fitzpatrick, 2014). According to previous studies, customers look up to celebrities and strive to replicate their lives, including cosmetics, clothing, fashion, restaurant choices, and even holiday locations. Businesses increasingly seek to include social media celebrities in their marketing strategies (Kumar and Mirchandani, 2012; Djafarova and Rushworth, 2017; Djafarova and Bowes, 2021). Fans of celebrities often seek advice. Consequently, it is thought that when celebrities post a message, the message’s authenticity will inspire followers to buy on impulse since individuals are prone to mimic celebrities’ actions (Wilcox and Stephen, 2013; Djafarova and Rushworth, 2017; Djafarova and Bowes, 2021). Homophily and attractiveness (i.e., social and physical) are characteristics of social media celebrities that establish attachment with followers.

Homophily is a sense of connectedness between relationship partners based on shared beliefs, interests, and memories (Chu and Kim, 2011; Kim and Kim, 2022). This notion refers to attitudinal similarity or shared subjective states toward a specific target, such as common interests, attitudes, and feelings (Kim and Kim, 2022). Celebrities may connect with fans by sharing commonalities. Similar lifestyles and personalities of social media superstars generate friendship and emotional connections (Ladhari et al., 2020; Kim and Kim, 2022). When social media superstars share personal stuff and get comments, fans feel closer to them. Followers are more engaged when they have comparable experiences, backgrounds, hobbies, value systems, or personal attributes. In this way, the homophily leads to more lively conversations, which creates emotional bonds (Chen et al., 2021).

In computer-mediated communication, social presence is “how much a medium permits the user to sense others as psychologically present” (Fulk et al., 1987). It means the ability to convey facial emotions, eye contact, nonverbal clues, and posture *via* a medium. Users’ sense of friendliness, warmth, personal relatedness, and media sensitivity may all contribute to the social presence (Short et al., 1976). Social presence may improve information quality by minimizing ambiguity and equivocation (Straub, 1994; Webster and Trevino, 1995) and increasing online conversation frequency and closeness (Straub, 1994; Jung, 2003; Rau et al., 2008). Moreover, in online transactions, social presence is essential for establishing customers’ confidence (Xu, 2014; Shan, 2016). Social presence is vital for social media superstars and fans. Social media superstars’ passionate and engaged communication boosts followership, trustworthiness, envy, and brand attitude (Djafarova and Rushworth, 2017; Jin et al., 2019; Djafarova and Bowes, 2021).

Physical attractiveness serves as an inferential signal for preliminary evaluation. People who are physically appealing may be considered integral, clever, and friendly (Joseph, 1982; Ali et al., 2021). Physical appearance may be critical to social bonding. Consumers are more attracted to human brands that are visually attractive (Cole and Leets, 1999; Ali et al., 2021). Social media superstars should be appealing (Djafarova and Rushworth, 2017; Ladhari et al., 2020; Djafarova and Bowes, 2021). When social media superstars reveal personal lives and connect with followers as human brands, attractiveness may attract attention and likeability. Attractive social media personalities may readily attract more followers, leading to emotional attachment.

According to Xiang et al. (2016), interacting with celebrities triggers impulsive behavior. In a social commerce context, homophily and social or physical attraction may trigger impulsive buying. Despite the rise of social networking sites, a recent comprehensive literature review found a scarcity of studies on impulsive purchase in social commerce, especially with context-specific stimulators (Xiang et al., 2016; Djafarova and Bowes, 2021). The above findings necessitate research into the moderating influence of digital celebrities’ phenomenon in impulsive buying on social commerce.

*H9*: Social media celebrity moderates the relationship between attitude and online impulse buying behavior.

## Materials and methods

We use a deductive approach in this research because hypotheses are developed based on previous research and theories (Saunders et al., 2007). The cross-sectional online survey was conducted to obtain data helpful in testing the model and examining the proposed hypothesis. This study has focused on China because China’s social media ecosystem is massive and mobile-focused. China has one billion active mobile social media users, the most in Asia-Pacific. China’s social media penetration rate increased to 68% in 2021, somewhat higher than the United States and Japan. Initially, data was gathered directly from the followers of digital personalities with a community of more than 75,000 members. Participants were asked to recall their most recent impulsive purchase prompted by digital celebrities on social media. A screening question was included to assure the process’s efficiency (do you recall your previous impulsive buy?). Thus, individuals without impulse-buying experience were excluded from the final sample. All constructs were measured using scales from previous well-established studies, as mentioned in Table 2. A seven-point Likert scale was used to operationalize all dimensions, with 7 indicating “strongly agree” and 1 indicating “strongly disagree.” The sample size is determined based on Comrey and Lee’s (2013) recommended criteria, suggesting a sample size of 50 as a poor, 300 as a good, 500 as a very good, and 1,000 is considered excellent. The ideal response rate for a consumer study questionnaire is between 40 and 60%, as Nulty (2008) suggested. Accordingly, we distributed 400 questionnaires and received 265 (66.25%) responses. For data analysis, 226 useable responses were gained after initial screening yielding a response rate of 56.5%. Respondents’ demographic profile is presented in Table 3.

**Table 2:** Measurement items.

Construct	Items	Code	Authors
Access Convenience	“Could shop anytime I wanted”	A-CON1	Jiang et al. (2013)
	“Could order products wherever I am.”	A-CON2	
	“The website is always accessible.”	A-CON3	
Search Convenience	“It was easy to navigate the website.”	S-CON1	Beauchamp and Ponder (2010)
	“The website provided useful information.”	S-CON2	
	“It was easy to get the information I needed to make my purchase decision.”	S-CON3	
Evaluation Convenience	“Provides detailed product specifications.”	E-CON1	Jiang et al. (2013)
	“Uses both text and graphics in the product information.”	E-CON2	
	“Sufficient information to identify different products”	E-CON3	
Transaction Convenience	“Flexible payment methods.”	T-CON1	Jiang et al. (2013)
	“My purchase was completed easily.”	T-CON2	
	“It did not take a long time to complete de purchase process.”	T-CON3	
Relationship Convenience	“The online retailer gave me personalized attention.”	R-CON1	Jun et al. (2004); Zahid et al. (2022)
	“The website had a message area for customer questions and comments.”	R-CON2	
	“I received a personal “thank you” note *via* email or other media after placing an order.”	R-CON3	
Possession convenience	“I got exactly what I wanted.”	P-CON1	Jiang et al. (2013); Beauchamp and Ponder (2010)
	“My order was delivered in a timely fashion.”	P-CON2	
	“Received all items I ordered.”	P-CON3	
Post possession Convenience	“It was easy to take care of returns and exchanges with the retailer.”	PP-CON1	Seiders et al. (2007)
	“X takes care of product exchanges and returns promptly.”	PP-CON2	
	“The retailer quickly resolves any after-purchase problems I experience.”	PP-CON3	
Cognitive attitude	‘Shopping on online shopping websites is effective.”	C-AAT1	Voss et al. (2003)
	“Shopping on online shopping websites is helpful.”	C-ATT2	
	“Shopping on online shopping websites is functional.”	C-ATT3	
Affective attitude	“Shopping on online shopping websites is exciting.’	A-AAT1	Voss et al. (2003)
	“Shopping on online shopping websites is delightful.”	A-ATT2	
	“Shopping on online shopping websites is enjoyable.”	A-ATT3	
Physical attractiveness	“Unattractive–attractive”	P-ATT1	McCroskey and McCain (1974); Ohanian (1990)
	“Ugly–beautiful”	P-ATT2	
	“Plain–elegant”	P-ATT3	
Social presence	“The influencer has a sense of sociality.”	S-PRE1	McCroskey and McCain (1974); Gefen and Straub (2004)
	“The influencer has a sense of human warmth”	S-PRE2	
	“The influencer provides a sense of human sensitivity”	S-PRE3	
Attitude homophily	“In general, the influencer who made the postings thinks like me”	A-HOM1	McCroskey and McCain (1974); Gilly et al. (1998)
	“In general, the influencer who made the postings behaves like me”	A-HOM2	
	“In general, the influencer who made the postings is similar to me”	A-HOM3	
Online Impulsive Buying Behavior	“During online shopping, I buy products without a lot of thinking.”	O-IBB1	Park et al. (2012)
	“I tend to buy things I have no desire to buy during online shopping.”	O-IBB2	
	“When I find something, I like on Instagram, I purchase it immediately”	O-IBB3	

**Table 3:** Demographic information.

Variables	Categories	Number	Percentage
Gender	Male	121	53.54
	Female	105	46.46
Age (in years)	18–20	106	46.90
	21–23	81	35.84
	23–25	39	17.26
Monthly family income (in RMB)	≤ 3,000	33	14.60
	3,001–5,000	103	45.57
	5,001–10,000	59	26.11
	>10,000	31	13.72

## Data analysis and results

A structural equation modelling – partial least square (PLS-SEM) approach was employed to analyze the data, and the SmartPLS (Ringle et al., 2015) software was used to do so. Osborne (2010) stated that statistical data in social science research generally have normality problems. PLS has a higher predictive ability while assessing complex theoretical models with small and big sample sizes and data that is not normally distributed (Hair Jr et al., 2017; Ali et al., 2021). In this study, data analysis is performed in two steps. The first step evaluates the measurement model, while the second step assesses the structural model. The measurement model examines the constructs’ reliability and validity, while the structural model tests the proposed hypothesized relationship (Becker et al., 2012; Ringle et al., 2015). In addition, a bootstrapping approach with 5,000 sub-samples was used to examine the *t*-values and level of significance for the path coefficient, as suggested by Hair et al. (2011).

### Measurement model

**Figure 1:**
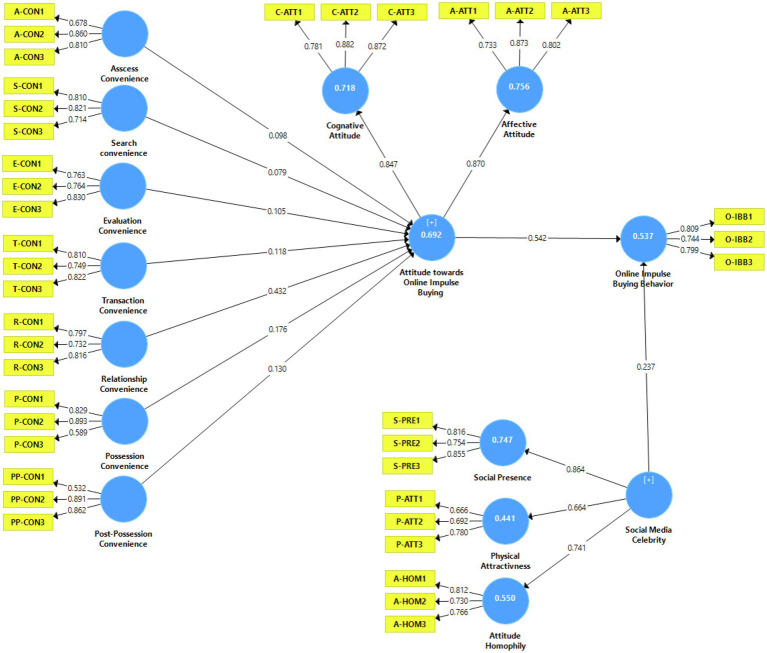
Measurement model.

The measurement model is assessed by determining the constructs’ reliability and validity. The reliability of the constructs is tested using composite reliability (CR). The results reported in Table 3 indicate that CR values lie between 0.757 and 0.883, showing that all constructs meet the threshold value criterion (i.e., 0.7; DeVellis, 2016). Similarly, constructs’ validity is examined using factor loadings and average variance extracted (AVE). All item loadings are above the value of 0.4–0.7, as suggested by Hair Jr et al. (2016). AVE values for all constructs range from 0.518 (Attitude) to 0.732 (Discomfort) and are above the recommended value of 0.5 (Fornell and Larcker, 1981; see Table 4 and Figure 1).

**Table 4:** Measurement model.

First order constructs	Second order constructs	Items	Loadings	CR	AVE
Access convenience		A-CON1	0.678	0.828	0.618
		A-CON2	0.860		
		A-CON3	0.810		
Search convenience		S-CON1	0.810	0.826	0.614
		S-CON2	0.821		
		S-CON3	0.714		
Evaluation convenience		E-CON1	0.763	0.829	0.619
		E-CON2	0.764		
		E-CON3	0.830		
Transaction convenience		T-CON1	0.810	0.836	0.631
		T-CON2	0.749		
		T-CON3	0.822		
Relationship convenience		R-CON1	0.797	0.826	0.613
		R-CON2	0.732		
		R-CON3	0.816		
Possession convenience		P-CON1	0.829	0.820	0.610
		P-CON2	0.893		
		P-CON3	0.589		
Post Possession convenience		PP-CON1	0.532	0.815	0.606
		PP-CON2	0.891		
		PP-CON3	0.862		
Cognitive-attitude		C-AAT1	0.817	0.883	0.716
		C-ATT2	0.811		
		C-ATT3	0.778		
Affective-attitude		A-AAT1	0.834	0.846	0.648
		A-ATT2	0.786		
		A-ATT3	0.774		
	Attitude	C-ATT	0.847	0.849	0.737
		A-AAT	0.870		
Social presence		S-PRE1	0.816	0.850	0.655
		S-PRE2	0.754		
		S-PRE3	0.855		
Physical attractiveness		P-ATT1	0.666	0.757	0.511
		P-ATT2	0.692		
		P-ATT3	0.780		
Attitude homophily		A-HOM1	0.812	0.813	0.593
		A-HOM2	0.730		
		A-HOM3	0.766		
	Social media celebrity	S-ATT	0.864	0.803	0.579
		P-ATT	0.664		
		A-HOM	0.741		
Impulsive buying Behavior		O-IBB1	0.809	0.828	0.616
		O-IBB2	0.744		
		O-IBB3	0.799		

Moreover, we employed the hetrotrait–monotrait ratios (HTMT) to evaluate the discriminant validity of instruments. The HTMT value must be below the suggested value of 0.85 or 0.9, as Kline (2015) recommended. All the constructs surpassed the threshold value (see Table 5). In addition, Variance inflation factor (VIF) values to examine the multicollinearity issues among constructs. Results showed that all the constructs have VIF values less than 3.3, indicating that multicollinearity is not a concern in this study (Kock and Lynn, 2012).

**Table 5:** Discriminant validity.

	A-ATT	A-CON	A-HOM	C-ATT	E-CON	O-IBB	P-ATT	P-CON	PP-CON	R-CON	S-CON	S-ATT	T-CON
A-ATT													
A-CON	0.566												
A-HOM	0.564	0.669											
C-ATT	0.623	0.543	0.578										
E-CON	0.599	0.493	0.580	0.560									
O-IBB	0.896	0.509	0.632	0.610	0.517								
P-ATT	0.537	0.410	0.384	0.409	0.443	0.432							
P-CON	0.621	0.465	0.424	0.626	0.455	0.434	0.641						
PP-CON	0.830	0.570	0.558	0.616	0.520	0.666	0.647	0.803					
R-CON	0.896	0.509	0.632	0.610	0.517	0.640	0.432	0.434	0.666				
S-CON	0.590	0.586	0.640	0.621	0.734	0.681	0.344	0.400	0.464	0.681			
S-ATT	0.863	0.581	0.606	0.692	0.606	0.839	0.566	0.600	0.641	0.839	0.566		
T-CON	0.791	0.534	0.512	0.612	0.591	0.694	0.668	0.680	0.804	0.694	0.557	0.865	

### Structural model

The overall model fitness was measured before proceeding to test the hypothesized relationship. Standardized root means square residual (SRMR) was used to determine the overall model fitness. The results indicate that the SRMR value is below the threshold value of 0.8, as suggested by Hu and Bentler (1998), indicating that model is a good fit. After determining the constructs’ validity and model fitness, the bootstrapping technique was considered with 5,000 resamples to test the significance level for path coefficients. In addition, t-test criterion at 95% confidence interval is used to examine the proposed relationship between independent and dependent variables (*t* > 1.645 and *p* < 0.05). The results show that all hypotheses are accepted (see Table 5). There is a significant and positive association between access convenience and attitude (*β* = 0.098, *t* = 3.147 > 1.64, *p* < 0.05). Similarly, search convenience (*β* = 0.079, *t* = 2.624 > 1.64, *p* < 0.05), evaluation convenience (*β* = 0.105, *t* = 3.338 > 1.64, *p* < 0.05), transaction convenience (*β* = 0.118, *t* = 3.222 > 1.64, *p* < 0.05), relationship convenience (*β* = 0.432, *t* = 13.555 > 1.64, *p* < 0.05), possession convenience (*β* = 0.176, *t* = 5.555 > 1.64, *p* < 0.05), and post-possession convenience (*β* = 0.130, *t* = 3.575 > 1.64, *p* < 0.05) significantly influences attitude. In addition, attitude has a significant and positive relationship with online impulse buying behavior (*β* = 0.542, *t* = 11.931 > 1.64, *p* > 0.05). According to Henseler and Sarstedt (2013), the predictive power of dependent variables can be used to determine the quality of the research model. Measures including the significance of path coefficient (*β*), coefficient of determination (*R*^2^), predictive relevance (*Q*^2^), and effect size (*f*^2^) were used to evaluate the model quality. The *R*^2^ score for online impulse buying behavior is 0.537, indicating that explanatory power is moderate, as per the suggestion of Hair et al. (2011). In addition, *Q*^2^ is used to assess the predictive relevance of the model. According to Hair et al. (2016), that model with a *Q*^2^ score above 0 is predictively relevant. The results demonstrate that the *Q*^2^ value of the proposed model is 0.295, indicating that model has the best predictive relevance. Moreover, Cohen (1988) proposed that *f*^2^ scores between 0.02, 0.15, and 0.35 have a small, medium, and large effect size. The proposed model’s *f*^2^ score justifies that the effect size lies between small and large (see Table 6).

**Table 6:** Hypotheses testing.

Hypothesis	Relationship	Path coefficient	Std. error	*t*-values	*p*-values	Results	*R* ^2^	*F* ^2^	*Q* ^2^
H1	Assess Convenience → Attitude	0.098	0.031	3.147	0.001	Supported	0.692	0.023	0.335
H2	Search Convenience → Attitude	0.079	0.030	2.624	0.004	Supported		0.013	
H3	Evaluation Convenience → Attitude	0.105	0.031	3.338	0.000	Supported		0.024	
H4	Transaction Convenience → Attitude	0.118	0.037	3.222	0.001	Supported		0.022	
H5	Relationship Convenience → Attitude	0.432	0.032	13.555	0.000	Supported		0.380	
H6	Possession Convenience → Attitude	0.176	0.032	5.555	0.000	Supported		0.067	
H7	Post-Possession Convenience → Attitude	0.130	0.034	3.757	0.000	Supported		0.029	
H8	Attitude → Online Impulse Buying behavior	0.542	0.045	11.931	0.000	Supported	0.537	0.295	0.314
	Moderating effect								
H9	Social media Celebrity*Attitude → Online Impulse Buyingbehavior	0.144	0.043	3.316	0.001	Supported	0.610	0.021	

### The moderation effect of social media celebrity

**Figure 2:**
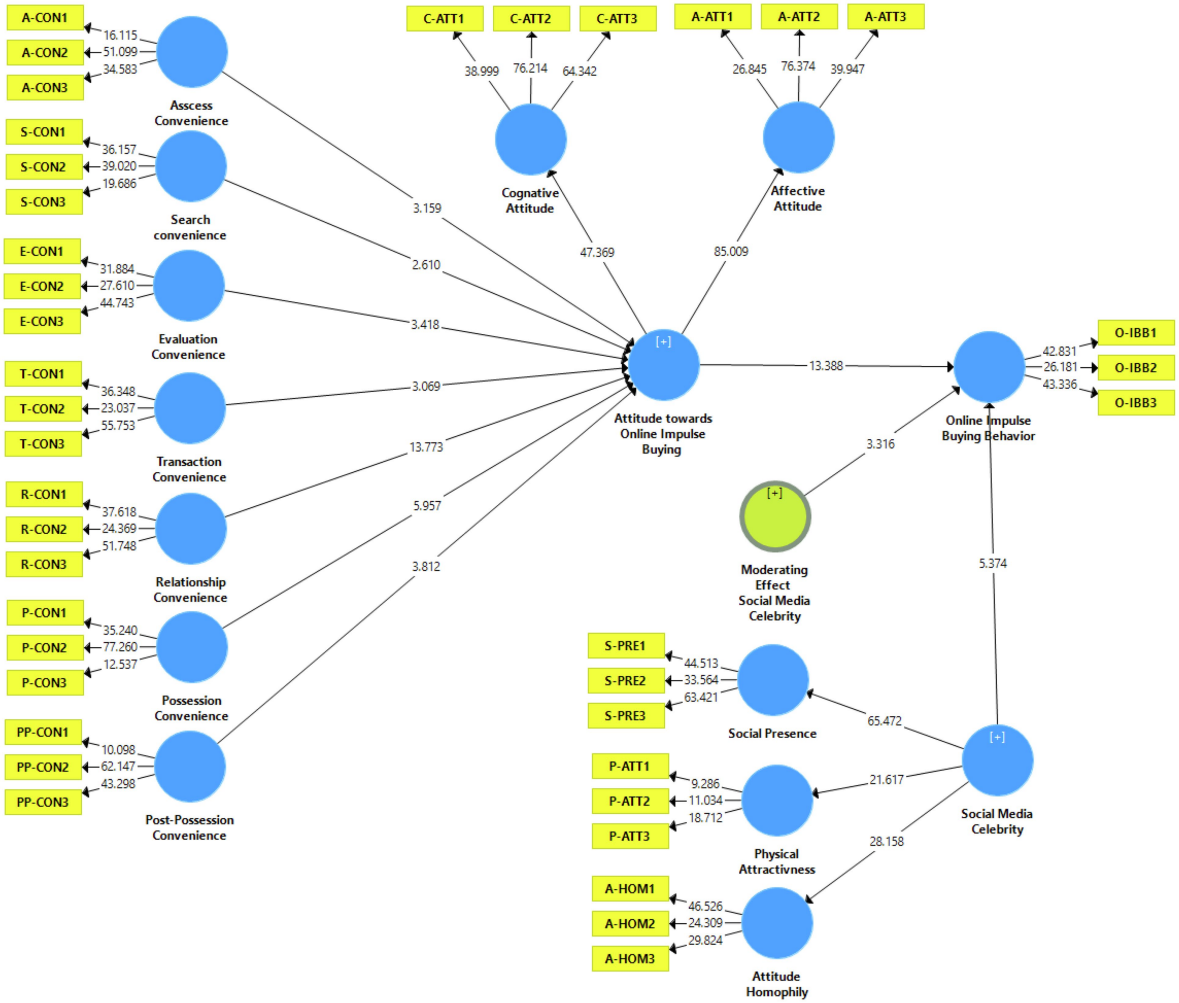
Moderating effect of social media celebrity.

The moderating effect of social media celebrity is investigated by testing its interaction effect on the relationship between attitude and online impulse buying behavior. The results demonstrate social media celebrity has a significant moderation effect on the relationship between attitude and online impulse buying behavior (*β* = 0.144, *t* = 3.316 > 1.96, *p* < 0.05), as mentioned in Table 6 and Figure 2. As a result of moderating effect of social media celebrity, *R*^2^ has increased from 0.537 to 0.610. After including social media celebrity in the model, the model’s the explanatory power has increased. However, the difference in change is small, but it is considered important in testing the moderation effect.

## Discussion and implications

### Discussion

By evaluating different dimensions of online service convenience, the recent research develops a comprehensive understanding of online service convenience as a stimulus for consumers’ online impulsive buying. We looked at seven key dimensions of online service convenience: access convenience, search convenience, evaluation convenience, transaction convenience, relationship convenience, possession convenience, and post-possession convenience. The study demonstrates that online convenience is a multidimensional construct with several dimensions. Service quality, individual consumer differences, and firm-related factors impact consumer perceptions of service convenience. Marketers can do much to enhance customers’ sense of convenience. They may often reduce customers’ time and effort costs and increase their happiness.

The examination of path coefficients showed that relationship convenience is the most critical factor influencing online convenience perception. Relationship convenience (= 0.86; *p* = 0.001) has emerged as the most important driver of online shopping convenience and is the primary reason people purchase online. The results are consistent with earlier research (Lin and Lu, 2010; Zahid et al., 2022).

Customers who shop online expect to get personalized attention and services that are better suited to their needs. This makes it easier and faster for them to find the information they need and decide what to buy. Possession has a significant influence on perceived online convenience. According to the present research, convenience is linked to “the speed and ease with which consumers can obtain desired products,” including production planning, stoking policies, shipping, and delivery timings. These results align with previous research (Islam, 2015; Aw, 2019). According to the findings, post-benefit convenience has a considerable impact on online impulsive purchase behavior. Because customers have had difficulty returning products acquired over the Internet in recent years, the results highlight the significance of post-possession ease. Retailers must handle refunds and exchanges. Retailers swiftly fix post-purchase issues.

Furthermore, the findings show that search convenience does not play a significant role in impulsive online purchases. These results align with (Shankar and Rishi, 2020; Jebarajakirthy and Shankar, 2021). Because all online retailers provide similar products and services, therefore search convenience does not affect buyers’ decisions. Furthermore, the findings show that access convenience has no impact on online impulsive purchase behavior. Previous research results contradict the findings of this study (Duarte et al., 2018; Jebarajakirthy and Shankar, 2021). Consumers may access internet services 24 h a day, 7 days a week, from any place, which might explain this finding.

The findings revealed a link between consumer attitude and behavior regarding impulsive online shopping. Other scholars have proposed a similar association (Kimiagari and Malafe, 2021). The results show that both dimensions of attitude impact online impulsive purchase behavior. Furthermore, the results show that consumers’ online impulsive purchase behavior is more closely linked to emotive rather than cognitive judgements. Consumers depend more on emotive judgments than cognitive judgments when making an online purchase choice. These results align with Kimiagari and Malafe’s (2021) findings. Finally, the moderated results show that social media superstars have a considerable impact on the attitude-behavior gap. Consumers are influenced by social media personalities, who encourage them to make impulsive purchases. The research findings show the effects of digital celebrities’ communities on impulsive behavior. The use of social media celebrities as influencers to reach customers outside of typical marketing tactics is supported by this research.

### Theoretical implication

From a theoretical standpoint, we make several additions to the existing literature. Providing excellent online services necessitates online convenience. As a result, various attempts have been undertaken to investigate the effect of online convenience on customer behavior (Jiang et al., 2013; Duarte et al., 2018; Pham et al., 2018). However, the influence of online convenience on customer response is still in its infancy in the online retailing context, and a comprehensive framework does not exist. Thus, investigating online convenience in the online retailing context is necessary. This research adds to the body of knowledge on how convenience influences customer behavior in a general and online retailing context in particular.

This research also proposes online convenience as a multidimensional first-order construct and investigates the relative influence of each dimension on impulsive buying. However, most studies in the existing literature addressed online convenience as a unidimensional or second-order construct, and its impact on consumer behavioral intention was investigated (Berry et al., 2002; Duarte et al., 2018; Wang et al., 2019). This research expands the online convenience literature by considering online convenience as a first-order construct.

Applying the classic S-O-R model to describe the influence of convenience factors on customer behavior is another scholarly contribution to this study. Previous studies lacked good theoretical foundations; this work adapts the S-O-R model to give a robust theoretical basis. By considering the stepwise process of predicting impulsive buying behavior, where online convenience dimensions were considered stimuli, attitudes as an organism, and buying behavior as the response, the operationalization of the S-O-R model provides better insights into online retailing literature. Second, this research reveals customers’ impulsive purchase behavior by incorporating a bi-dimensional attitude approach into the structural model. The study helps to better understand customers’ rational and emotional judgments of impulsive online shopping by integrating cognitive and affective attitudes.

Finally, the moderating effects of social media celebrity on the relationship between attitude and behavior were explored, which is uncommon in the current research on online impulsive purchase behavior. As a result, this study proposes a thorough moderated model to investigate the influence of online convenience on customer behavior toward impulsive buying.

### Practical implications

Online merchants may use the online shopping convenience model as a diagnostic tool to determine which convenience dimensions and associated aspects are most important to their consumers. From a management standpoint, the results give managers a better understanding of which aspects of convenience they should concentrate on to improve total online convenience. Hence, they enhance customer satisfaction and e-WOM. The results also help retailers in online shopping convenience management. Customers participate in online shopping for relationship, possession, and post-possession convenience. Retailers should be aware of these three factors.

Complementary activities should next be considered since Chinese online shoppers are worried about the difficulty of returning an item or receiving a refund. Investing in new methods to earn online consumers’ confidence and compensate them for a bad deal might be crucial in enhancing online convenience, contentment, and readiness to use and refer to the service. These may be achieved by paying particular attention to the packaging of products to prevent damage during transit and the delivery location and time, warranty, and return policy. The present results point to the necessity for firms to ensure that expectations and actual performance are consistent. Other tips for Chinese online shopping companies include offering thorough product information and delivering effective customer service during and after the online transaction.

Furthermore, due to the enormous power of social media superstars, companies should use them for marketing their products. For example, Marketers may provide complete product information to digital superstars and invite them to do a live product review. Aside from that, the current research gives valuable results that show social media celebrity as a relevant channel for advertising and promoting a business and a marketing communication tool that impacts the shopping process.

### Limitations and future research directions

The study includes a few flaws that might be addressed in future research. Because the investigation is confined to China, future studies might replicate it in other countries to further generalize the results. Furthermore, technology is constantly evolving, leading to technophobia. Therefore, longitudinal research is necessary to investigate the influence of online convenience. Furthermore, the proposed model might be used to examine the impact of online convenience on purchasing behavior in different online shopping situations, such as online shopping for luxury goods.

## Data availability statement

The raw data supporting the conclusions of this article will be made available by the authors, without undue reservation.

## Author contributions

All authors listed have made a substantial, direct, and intellectual contribution to the work and approved it for publication.

## Conflict of interest

The authors declare that the research was conducted in the absence of any commercial or financial relationships that could be construed as a potential conflict of interest.

## Publisher’s note

All claims expressed in this article are solely those of the authors and do not necessarily represent those of their affiliated organizations, or those of the publisher, the editors and the reviewers. Any product that may be evaluated in this article, or claim that may be made by its manufacturer, is not guaranteed or endorsed by the publisher.
